# 

**Published:** 1890-06

**Authors:** 


### WILLIAM F. JENKS MEMORIAL PRIZE.

*The Second Triennial Prize, of Four Hundred and Fifty Dollars, under the Deed of Trust of Mrs. William F. Jenks, will be awarded to the author of the best essay on*

"THE SYMPTOMATOLOGY AND TREATMENT OF THE NERVOUS DISORDERS FOLLOWING THE ACUTE INFECTIOUS DISEASES OF INFANCY AND CHILDHOOD."

THE conditions annexed by the founder of this prize are, that the "prize or award must always be for some subject connected with Obstetrics, or the Diseases of Women, or the Diseases of Children;" and that "the Trustees, under this deed for the time being, can, in their discretion, publish the successful essay, or any paper written upon any subject for which they may offer a reward, provided the income in their hands may, in their judgment, be sufficient for that purpose, and the essay or paper be considered by them worthy of publication. If published, the distribution of said essay shall be entirely under the control of said Trustees. In case they do not publish the said essay or paper, it shall be the property of the College of Physicians of Philadelphia."

The prize is open for competition to the whole world, but the essay must be the production of a single person.

The essay, which must be written in the English language, or if in a foreign language, accompanied by an English translation, should be sent to the College of Physicians of Philadelphia, Pennsylvania, U.S.A., before January 1st, 1892, addressed to Louis Starr, M.D., Chairman of the William F. Jenks Prize Committee.

Each essay must be distinguished by a motto, and accompanied by a sealed envelope bearing the same motto and containing the name and address of the writer. No envelope will be opened except that which accompanies the successful essay.

The Committee will return the unsuccessful essays if reclaimed by their respective writers, or their agents, within one year.

The Committee reserves the right not to make an award if no essay submitted is considered worthy of the prize.

Bristol Medico-Chirurgical Journal.

JUNE, 1890.

*Cases for binding, price 7d. each, may be had of the Publisher,*
J. W. ARROWSMITH, *Quay Street, Bristol.*

### PUBLICATIONS RECEIVED.

From John Bale & Sons, London:
*On the Simulation of Hysteria by Organic Disease of the Nervous System.* By Thomas Buzzard, M.D. 1890. Reprint.
*On Vertigo of Bulbar Origin.* By Thomas Buzzard, M.D. 1890. Reprint.
From T. B. Burrow, London:
*Hand and Heart.* June, 1890.
From Ev. E. Carreras, St. Louis:
*The Alienist and Neurologist.* April, 1890.
From J. & A. Churchill, London:
*The Palliative Treatment of Incurable Cancer.* By Herbert Snow, M.D. 1890.
*Re-Appearance of Cancer after Operation.* By Herbert Snow, M.D. 1890.
From William Clowes & Sons, Limited, London:
*Unsoundness of Mind.* By J. W. Hume Williams. 1890.
From F. A. Davis, Philadelphia:
*Practical Electricity in Medicine and Surgery.* By G. A. Liebig, Jr., Ph.D., and George H. Rohé, M.D. 1890.
*A System of Practical and Scientific Physiognomy.* By Mary Olmsted Stanton. Two volumes. 1890.
From Octave Doin, Paris:
*De la Mobilisation de l'Etrier.* Par le Docteur E. J. Moure. 1890.
From Feret, Bordeaux:
*Annales de la Polyclinique.* January, 1890.
From G. Gounouilhou, Bordeaux:
*De la Grippe et de son traitement par le Sulfate de Quinine.* Par le Dr. P. Gellie. 1890.
From Charles Griffin and Company, London:
*The Surgery of the Kidneys.* By J. Knowsley Thornton. 1890.
*Clinical Diagnosis.* By Dr. Rudolf v. Jaksch. 1890.
From J. N. Jaros, New York:
*Coca and its Therapeutic Application.* By Angelo Mariani. 1890.
From H. K. Lewis:
*The Extra Pharmacopœia.* By William Martindale. Sixth Edition. 1890.
*Chronic Urethritis.* By Matthew Berkeley Hill, M.B. 1890.
*The British Journal of Dermatology.* April—June, 1890.
*Disease in Children.* By Angel Money, M.D. Second Edition. 1890.
*Diseases of Women.* By Arthur H. N. Lewers, M.D. Second Edition. 1890.
*Practical Chemistry.* By Samuel Rideal, D.Sc. 1890.
*Flushing and Morbid Blushing.* By Harry Campbell, M.D. 1890.
*Dental Surgery.* By A. W. Barrett, M.B. Second Edition. 1890.
From Willis McDonald & Co., New York:
*The Dietetic Gazette.* April, 1890.
From John P. Morton and Company, Louisville:
*The Four Commencements.* By J. M. Bodine, M.D. 1890.
From Young J. Pentland, Edinburgh:
*Studies in Clinical Medicine.* By Byrom Bramwell, M.D. Nos. 19, 20. 1890.
From John Morgan Richards, London:
*Medical Reprints.* Nos. 2—4. 1890.
From Stillwell and Co., Melbourne:
*Intercolonial Medical Congress of Australasia. Transactions of Second Session, held in Melbourne, Victoria, January,* 1889. 1889.
From Smith, Elder & Co., London:
*Curvatures of the Spine.* By Noble Smith. Third Edition. 1889.
From John Wright & Co., Bristol:
*Workhouse Medical Officer.* By Alfred Sheen, M.D. Second Edition. 1890.
From 36 Corso Porta Romana, Milan:
*Sulla Efficacia Terapeutica della Catramina Bertelli nelle Tubercolosi Locali ed Esplicitazioni Morbose Affini.* Osservazioni Cliniche del. Cav. Uff. Dott. Fernando Franzolini.. 1890.
From 126 East 59th Street, New York:
*Twenty-second Annual Report of the New York Orthopædic Dispensary.* 1890.
From 59 Maiden Lane, New York:
*The International Journal of Surgery.* May, 1890.
From 3 Rue Christine, Paris:
*Revue chirurgicale.* Nos. 6—11. 1890.

